# Effect of pimobendan on physical fitness, lactate and echocardiographic parameters in dogs with preclinical mitral valve disease without cardiomegaly

**DOI:** 10.1371/journal.pone.0223164

**Published:** 2019-10-03

**Authors:** Nayeli Iwanuk, Leona Wall, Ingo Nolte, Jonathan Raue, Katja Rumstedt, Anna Pilgram, Maximiliane Sehn, Karl Rohn, Jan-Peter Bach

**Affiliations:** 1 Small Animal Clinic, University of Veterinary Medicine Hannover, Foundation, Hannover, Germany; 2 Institute of Biometry, Epidemiology and Information Processing, University of Veterinary Medicine Hannover, Foundation, Hannover, Germany; Auburn University College of Veterinary Medicine, UNITED STATES

## Abstract

Pimobendan has gained enormous importance in the treatment of mitral valve disease in dogs. The current consensus statement of the American College of Veterinary Internal Medicine (ACVIM) recommends a treatment for dogs with symptomatic disease and dogs with asymptomatic disease with radiographic and echocardiographic signs of cardiomegaly. To investigate whether these dogs also benefit from a therapy with pimobendan, 21 dogs with mitral valve disease ACVIM B1 underwent a standardized submaximal exercise test on a treadmill. In this double-blinded and randomized study, the animals were divided into two groups, one receiving pimobendan and the other a placebo. At the first visit and at every follow-up appointment (at days 90 and 180), heart rate during the complete exercise test and lactate before and after running were measured. In addition to this, a questionnaire was completed by the dogs’ owners and all dogs were given an echocardiographic examination to detect any changes and to observe if the disease had progressed. Due to the diagnosis of leishmaniosis, one dog in the pimobendan group was retrospectively removed from the study so that 20 dogs were included for statistical analysis. No differences were observed at any time between the pimobendan-group and the placebo-group regarding heart rate. At day 180, the increase in lactate after exercise was significantly lower than in the placebo-group. The increase in the pimobendan-group at day 180 was lower than at day 90. Most of the dog owners from the pimobendan-group declared that their dogs were more active at day 90 (6/10) and at day 180 (8/10), while most dog owners from the placebo-group observed no changes regarding activity at day 90 (8/10) and day 180 (6/10). It can be concluded that the results of this study indicate that some dogs with mitral valve disease ACVIM B1 might benefit from a therapy with pimobendan.

## Introduction

Mitral valve disease is a progressive disease which can cause congestive heart failure and sudden death [1]. The disease is very common among dogs, especially among dogs of smaller breeds [1, 2]. The American College of Veterinary Internal Medicine (ACVIM) consensus statement currently recommends initiating therapy in stage B2 (asymptomatic disease with radiographic and echocardiographic signs of cardiomegaly) [3].

So far, there have been few reports regarding treatment of dogs in stage B1 (asymptomatic disease without cardiomegaly); evidence for a positive treatment effect is lacking and treatment is not recommended [3]. There have even been some reports regarding the advance of structural cardiac changes after administering pimobendan in healthy dogs and in dogs with early stage mitral valve disease [4–6]. Neither the report by Chetboul et al. nor the report by Tissier et al. made any statements regarding effects of the medication on survival time, time to onset of clinical symptoms or quality of life. The latter, however, is one of the most important criteria for dog owners when they are considering therapeutic options for their pets [7]. Questionnaires, like the functional evaluation of cardiac health score (FETCH-score), are common for objectively assessing the quality of life of dogs with heart failure and other diseases [8–10].

In conclusion, there is still a lot of uncertainty regarding the treatment of dogs with asymptomatic mitral valve disease without signs of cardiomegaly (ACVIM B1), while a positive effect of treatment with pimobendan for ACVIM B2 and ACVIM C patients is well established.

Although exercise tests are commonly used in human cardiac patients, their use in small animal medicine has mainly been limited to research purposes [11–15]. This may be due to the lack of a standardized objective testing method and additional limitations like the strong physiological variance in dogs due to different breeds and different sizes [12]. Still, their potential for veterinary cardiology has been established in different research projects [12, 15, 16]. Covered distance and lactate are indicators of exercise tolerance and fitness level [12, 14]. Kittleson et al. observed that while exercising, blood lactate increases earlier in dogs with heart failure than in those without cardiac diseases [16]. Since lactate is a product of anerobic metabolism [17, 18], a higher lactate-value in dogs with heart failure indicates a lower oxygen concentration in the tissue. Wall et al. observed that lactate-values tended to be higher in dogs with mitral valve disease ACVIM B than in healthy dogs after exercise [15]. Exercise tests are a suitable tool for examining the effect of medication on exercise tolerance and have been used to assess the effect of pimobendan in humans in the past [19–20].

The aim of the current study was to investigate the influence of pimobendan regarding exercise tolerance, fitness and quality of life in dogs with early mitral valve disease (ACVIM B1). Exercise tests were used in conjunction with measurements of lactate to observe the effect of pimobendan on physical fitness. In addition, dog owners were interviewed using the FETCH-Score regarding changes in activity and physical endurance in their dogs to determine an influence of pimobendan on physical fitness and quality of life. To investigate the effects of pimobendan on cardiac function or morphology as well as any delay in the progression of the disease, cardiac ultrasound was performed.

## Materials and methods

### Study design

This study was a prospective, double-blinded, randomized, placebo-controlled study. It was approved by an ethical review committee (Lower Saxony State Office for Consumer Protection and Food Safety (LAVES), 33.9-42502-05-14A484) and every dog owner signed a declaration of agreement.

### Patient selection

Dogs with mitral valve disease Stage ACVIM B1 (presymptomatic disease without signs of cardiomegaly or congestive heart failure) were eligible for participation in the study. Dogs had to have a typical heart murmur, valvular lesions on the mitral valve and mitral regurgitation identified through echocardiographic examination. They were required to have no signs of congestive heart failure or left atrial or ventricular enlargement. Left atrial and ventricular size were assessed by the ratio of diameters of left atrium and aortic root (LA/Ao ratio, ≤ 1.6) and the diastolic diameter of left ventricle (LVIDDn, ≤1.7) during echocardiographic examination. The LA/Ao ratio was measured in accordance with Hansson et al. [21]. The LVIDDn was calculated using the following formula: LVIDDn = LVIDd(cm)/(BW(kg))^0.294^ [22]. The vertebral heart score (VHS) [23] had to be within the reference range for the examined breed, but was only analyzed if an x-ray picture was available. Dogs with severe systemic diseases or orthopedic symptoms and those unwilling to run on the treadmill were excluded from the study. In addition to the examinations described below, cardiac biomarker testing was also performed in conjunction with exercise testing in the dogs participating in the study [24].

### Schedule of events

All examinations were performed at the initial clinic visit and at follow-up appointments at days 90 and 180. Patient history was recorded and every dog underwent a detailed clinical examination. Blood samples were collected to perform a complete blood count and a clinical chemistry to rule out non-cardiac diseases. To classify the dogs into the appropriate stages of the ACVIM system, an echocardiographic examination (Vivid E9, GE Healthcare GmbH, Solingen, Germany) was performed. Among other parameters, fractional shortening (FS %), LVIDD and LA/Ao ratio were measured and the Cornell-Index of LVIDD (LVIDDn) was calculated. Before running on the motorized treadmill (“quasar”, h/p/cosmos sports and medical GmbH, Nussdorf-Traunstein, Germany), an electrocardiogram was performed for ten minutes to rule out possible arrhythmia. Before and after running on the treadmill, blood was sampled to measure the lactate levels (Cobas c 311 Analyser Roche Diagnostics GmbH, Mannheim, Germany), electrolytes (Rapidlab 1260, Siemens Healthcare Diagnostics GmbH, Eschborn, Germany) and acid-base state (Rapidlab 1260, Siemens Healthcare Diagnostics GmbH, Eschborn, Germany). Lactate was measured from sodium-flouride-plasma samples by cobas c 311 analyser (Roche Diagnostics, Mannheim / Germany) in the first ten minutes after drawing blood. In-house validation of all used laboratory tests was performed prior to the beginning of the study and repeated on a regular basis.

### Blinding, randomization and allocation

Dogs were assigned to either pimobendan or placebo groups by drawing lots. Two blocks of ten lots (five for pimobendan,and five for placebo-group) were prepared for the first 20 dogs included in the study. The last dog was randomly assigned to the pimobendan-group by drawing lots as well. Group allocation was performed by a person who had no other role in the study. This person also held the randomization data for the duration of the study until completion of statistical analysis.

### Trial medication

The trial medication of pimobendan was administered by the owners at a dose of 0.4 milligram per kilogram per day (mg/kg/d), divided into two administrations given every 12 hours, always one hour before feeding. The placebo contained milk sugar and looked similar to the pimobendan capsules. No other cardiac treatment was administered by veterinarians or owners for the duration of the study.

### Standardized, submaximal exercise test

In the current study, the standardized submaximal exercise test established by Wall et al. was used [15]. Before the submaximal exercise test started, there was a short familiarization period in which dogs learned to run on the treadmill and in which their individual speed was detected. Dogs had to trot on the treadmill, being motivated to do so by food or toys. The mean speed and the standard deviation (SD) was 5.5 (± 1.3) kilometers per hour (km/h). Every dog ran at its own comfort speed (ranging from 3 km/h to 7km/h), but at every follow-up appointment the dogs ran at the same speed as at the first appointment. The standardized, submaximal exercise test included six stages. Each stage lasted three minutes and after each stage, the incline was increased by 4%. The initial incline on the treadmill was 0% and the maximal incline in stage 6 was 20%. There was a 20-second break after stages 1, 3 and 5 and a three—minute break after stages 2 and 4. The heart rate was measured continuously by a Polar heart rate monitor (Polar FT7N and Polar H1, Polar Electro GmbH Deutschland, Büttelborn / Germany). Five consecutive heart rate values were recorded during the final 30 seconds of each stage of the exercise test. The average of these five values was calculated and recorded as the heart rate value for that stage. Before running, after running and five minutes after finishing the submaximal exercise test, the respiratory frequency and the rectal body temperature were measured. If the dog showed any sign of overstraining or if the heart rate was 240 beats per minute (bpm) or higher, the exercise test was stopped immediately. To ensure a sufficient workload, the heart rate had to increase by 40% compared to the heart rate at rest.

### Questionnaire

A questionnaire orientated on the FETCH-score was used [9]. At the follow-up appointments at days 90 and 180, dog owners had to evaluate possible changes in activity and physical endurance of their dogs in the previous 90 days. No additional criteria for evaluating the dogs’ activity and endurance were given and classification was left to the subjective assessment of the dogs’ owners. The full questionnaire can be found in the supplementary material (S1 Questionnaire).

### Statistical methods

The assessment of the model residues for normal distribution was done by visual assessment of the qq-plots and the Shapiro-Wilk-test. The assumption of normal distribution was not rejected. A three-way analysis of variance-covariance (ancova) with independent and repeated measurements and age and weight as continuous covariables, taking into account the interaction term of effects, was calculated for the lactate values and heart-rate for both groups at any time before and after running on the treadmill. The post-hoc tukey-test was calculated for multiple pairwise comparisons, taking into consideration the experimentwise error rate (EER). In the same way, a two-way analysis of variance was used for measuring the cardiac ultrasound (FS, LVIDDn, LA/Ao ratio), for which no measurements were carried out after exercise testing. The findings of the questionnaire regarding activity and physical resilience as dichotomous variables in the two groups at day 90 and day 180 day were calculated with the Fisher’s exact test. Statistical analysis was performed using commercial software SAS 9.4 with the Enterprise Guide Client 7.15 (SAS Institute Inc., Cary, NC, USA). A p-value of <0.05 was considered to be significant.

## Results

### Base-line characteristics

Twenty-five client-owned dogs of different breeds were enrolled for the study. Four of these dogs had to be excluded due to chronic lameness (n = 1), liver carcinoma (n = 1) or complete reluctance to run on the treadmill after initial examinations (n = 2). Twenty-one dogs completed the study. One of these dogs was diagnosed with leishmaniosis after the study was completed and had to be retrospectively removed from the study. Twenty dogs (ten in the pimobendan-group and ten in the placebo-group) were included in the final analysis. Table 1 shows details regarding the included dogs. There were no significant differences between the two groups of dogs regarding age, weight or heart murmur intensity. Table 2 shows the distribution of abortion of the submaximal exercise test and the number of dogs which completed all test stages.

**Table 1:** Breed, gender, age, weight, heart-murmur and running speed on the treadmill of each included dog.

number	breed	gender	age (years)	weight (kg)	heart murmur	speed (km/h)
**1**	mixed-breed	mn*	11.1	31.6	II/VI	5.5
**2**	mixed-breed	mn*	10.8	16.6	I/VI	5.5
**3**	mixed-breed	mn*	9.8	16.0	I/VI	6.3
**4**	mixed-breed	fn**	10.8	26.6	I/VI	6.4
**5**	mixed-breed	fn**	15.7	8.7	IV/VI	4.0
**6**	mixed-breed	fn**	7.7	11.9	II/VI	4.5
**7**	mixed-breed	fn**	6.2	6.8	I/VI	4.4
**8**	Golden Retriever	fn**	8.5	34.5	I/VI	7.0
**9**	Golden Retriever	mi***	8.1	28.7	II/VI	6.5
**10**	Jack Russel Terrier	mn*	13.5	12.0	IV/VI	3.0
**11**	Jack Russel Terrier	mn*	12.4	7.6	II/VI	4.1
**12**	Beagle	fn**	7.9	18.8	II/VI	7.0
**13**	Beagle	mn*	6.2	18.8	IV/VI	6.3
**14**	Cavalier King Charles Spaniel	fn**	6.8	8.0	IV/VI	5.5
**15**	Entlebuch cattle dog	fn**	5.8	18.0	I/VI	6.0
**16**	Small Munsterlander	fn**	11.8	19.8	I/VI	6.7
**17**	Pekinese	mn*	6.2	8.9	I/VI	3.4
**18**	West Highland White Terrier	fn**	7	6.9	I/VI	3.0
**19**	Poodle	mn*	12.2	10.6	II/VI	4.5
**20**	American Staffordshire Terrier	mn*	7.8	16.5	I/VI	5.3

*mn: male neutered

**fn: female neutered

***mi: male intact.

Ten female dogs (all neutered) and ten male dogs (one intact, nine neutered) completed the study and were included in the statistical analysis. Mean age was 8.3 (± 2.8) years and mean weight was 16.5 (± 8.3) kg. Heart murmur intensity was graded from 1–6, using the Levine scale.

**Table 2:** Distribution of the number of dogs aborting or completing the submaximal exercise test.

	number of abortions						all stages completed	total number
	stage 1	stage 2	stage 3	stage 4	stage 5	stage 6		
**day 0**	0	0	1	1	1	0	17	20
**day 90**	0	0	0	2	1	0	17	20
**day 180**	1	0	0	0	2	1	16	20

Distribution of the number of dogs aborting and the number of dogs completing all of the six stages of the submaximal exercise test at days 0, 90 and 180. The total number of dogs is written on the right side. The reasons for aborting the exercise test were lameness (n = 1) or reluctance to run (n = 9). The heart rate of the dogs did not exceed 240 bpm at any point of the exercise test and no dog showed signs of overstraining.

### Lactate

The following table shows the lactate-values (mg/dl) before and after exercise and increases in the lactate levels in the the pimobendan-group and the placebo-group (Table 3) at days 0, 90 and 180.

**Table 3:** Lactate-levels (mg/dl; mean ± SD) before and after exercise and mean (± SD) increases in lactate levels (mg/dL) after exercise in the pimobendan- and placebo-group.

		day 0	day 90	day 180
**Pimobendan-group**	before exercise	13.7 (± 4.3)	14.5 (± 3.5)	16.2 (± 3.0)
	after exercise	16.0 (± 5.9)	16.5 (± 4.3)	13.7 (± 3.6)
	increase	2.3 (± 7.0)	2.0 (± 3.7)	-2.5 (± 3.7)
**Placebo-group**	before exercise	12.8 (± 5.4)	13.2 (± 3.6)	11.0 (± 2.7)
	after exercise	13.9 (± 5.5)	14.9 (± 4.9)	13.3 (± 4.7)
	increase	1.1 (± 4.3)	1.7 (± 5.3)	2.3 (± 4.1)

**Fig 1:**
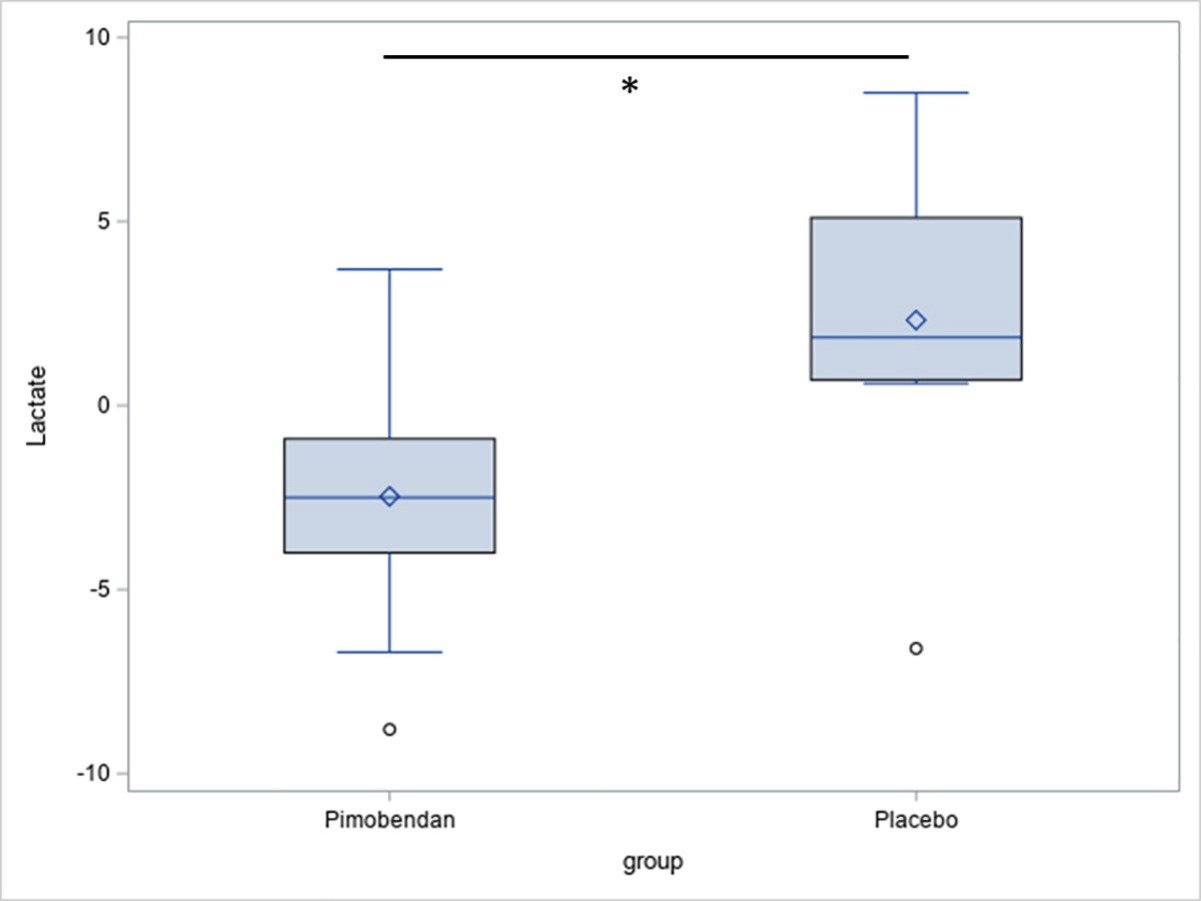
Increase in lactate levels (mg/dL) after exercise–comparison of groups. There was a significant difference (*) between the increase in lactate in the pimobendan- and placebo-group at day 180 (p = 0.011).

**Fig 2:**
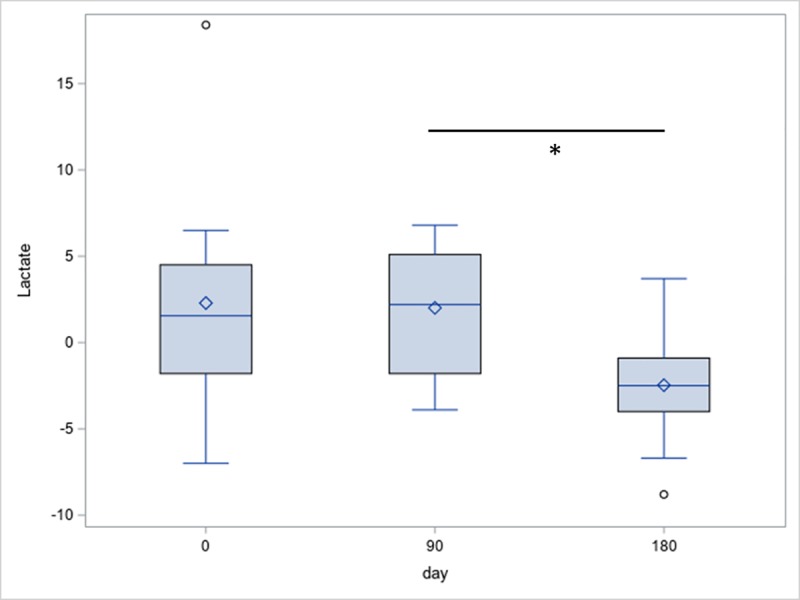
Increase in lactate levels (mg/dL) after exercise within the pimobendan-group. There was a significant difference between the increase at days 0 and 180 in the pimobendan-group (p = 0.033).

There was no significant difference regarding the lactate values between the pimobendan- and placebo-group at any point. There was also no significant difference between the initial examination and follow-up examinations in either group. At day 180, the increase in lactate levels after exercise in the pimobendan-group was significantly lower than in the placebo-group (p = 0.011; Fig 1). Actually, the lactate value after exercise decreased in the pimobendan group in comparison to the value before exercise. In the pimobendan-group the change of lactate after exercise is significantly lower on day 180 than on day 90 (p = 0.033; Fig 2).

### Questionnaire

**Fig 3:**
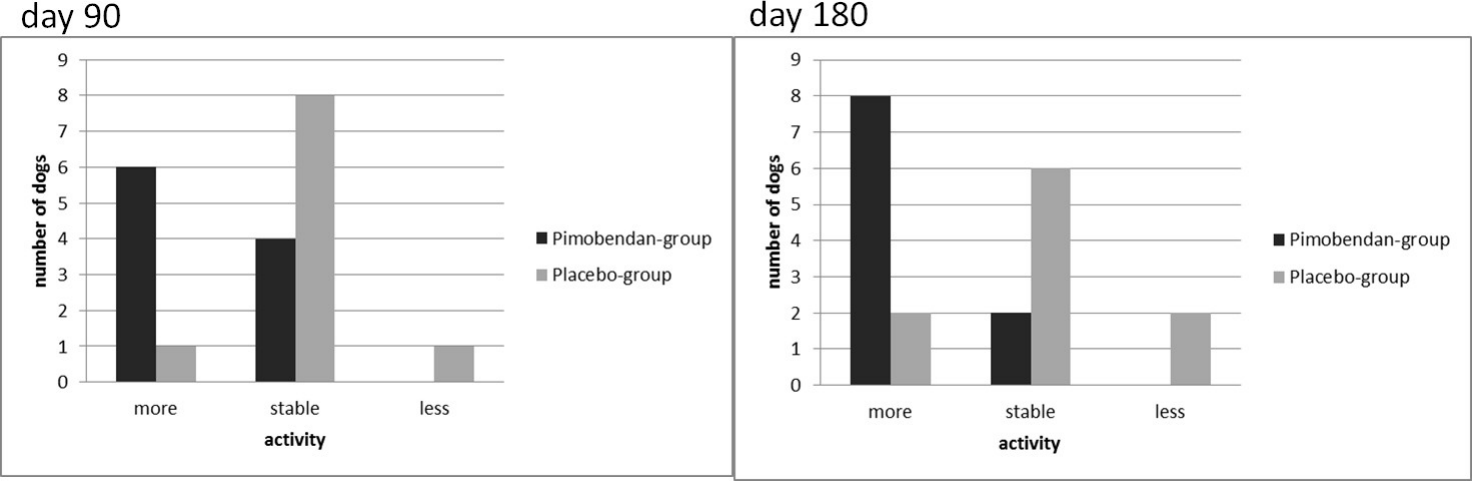
Changes in the dogs’ activity (more, stable, less) evaluated by patient owners. The number of dogs is labeled on the y-axis and the changes during 90 or 180 days of administering medication (more, stable, less) are labeled on the x-axis. The pimobendan-group is marked in black and the placebo-group in gray.

Fig 3 shows the evaluation of the change in activity by patient owners of their dogs after 90 and 180 days of administering pimobendan or the placebo. More dogs were described as being more active in the pimobendan-group at both evaluation times. The difference between the pimobendan- and placebo-group regarding the dogs’ physical activity was not significant at day 90 (p = 0.057) but at day 180 (p = 0.033). There was no significant difference regarding the dogs’ activity between days 90 and day 180 in either group. No negative side-effects were detected by the patient owners. There were no significant differences in physical endurance in either group at any time.

### Cardiac ultrasound

There was no significant difference in the LA/Ao-ratio, in the LVIDDn or in the FS between the groups at any point. There was also no significant difference between the three points of measurement in these parameters in either group.

Table 4 shows the LA/Ao-ratio, the LVIDDn and fractional shortening (FS %) of the dogs at days 0, 90 and 180 of administering pimobendan and the placebo.

**Table 4:** Echocardiographic parameters LA/Ao-ratio (mean ± SD) LVIDDn using the Cornell-Index (mean ± SD), FS (mean ± SD) of the dogs and the p-values for comparing the pimobendan- and placebo-group at the different examination times.

	Pimobendan-group	Placebo-group	p-value
	LA/Ao		
**day 0**	1.22 (± 0.17)	1.28 (± 0.12)	0.35
**day 90**	1.26 (± 0.12)	1.31 (± 0.16)	0.39
**day 180**	1.28 (± 0.13)	1.37 (± 0.17)	0.22
	LVIDDn		
**day 0**	1.57 (± 0.13)	1.62 (± 0.14)	0.37
**day 90**	1.46 (± 0.22)	1.54 (± 0.15)	0.38
**day 180**	1.48 (± 0.25)	1.57 (± 0.17)	0.41
	FS		
**day 0**	34.66 (± 9.10)	32.67 (± 11.62)	0.68
**day 90**	41.49 (± 8.69)	36.47 (± 7.64)	0.19
**day 180**	38.16 (± 10.13)	34.28 (± 8.19)	0.31

### Heart rate

There was a significant difference in heart rate before and after exercise (p<0,001). There was no significant difference in the heart rate between the groups at any point (day 0 p = 0.083, day 90 p = 0.22, day 180 p = 0.39, respectively). Also, there was no significant difference between days 0, 90 and 180 in either group.

## Discussion

The aim of the current study was to investigate the influence of pimobendan on patients with early mitral valve disease (ACVIM B1) and the applicability of combined exercise testing and measurement of lactate levels to evaluate the effect of pimobendan on physical fitness and quality of life. In addition to this, dog owners were questioned by the FETCH-score whether there were any changes in activity or physical endurance [9]. Another objective was to investigate whether pimobendan had any effects on cardiac function, morphology or on heart rate.

As stated in a previous study, there is clear evidence that dogs with mitral valve disease stage ACVIM B2 benefit from treatment with pimobendan [7]. Nevertheless, it is still unclear if the aforementioned therapy is beneficial for dogs with mitral valve disease stage ACVIM B1. Several parameters were examined in this double-blinded and randomized study to help answer this question. No detrimental effects of pimobendan therapy were observed in patients in this study and many dog owners reported an increased activity of their dog after administering pimobendan.

### Lactate

Exercise on a treadmill results in an increase in lactate-values in dogs [14, 18]. Values measured under exercise conditions are indicators of the fitness level of dogs [12, 25]. In the current study, a significant difference in increase after exercise between the groups at day 180 was observed. The pimobendan-group showed a decrease and the placebo-group an increase after exercise, compared to the other time points. The difference in the increase between the groups shows that it is important to measure lactate-values after exercise and to observe the increase after exercise. There were no differences between the groups before exercise, but the basic lactate-values in the placebo-group tended to be lower than in the pimobendan-group at day 180. Possibly, dogs in the pimobendan-group exerted more physical activity than dogs in the placebo-group shortly before the appointment in the clinic. If this had been the case, lactate-values would have been higher because of the exercise before drawing blood. In addition to this, the difference was not significant. Thus, standardized exercise tests are important to detect and compare changes in lactate values and consequently in oxygen concentration in the tissue. These findings coincide with the observation by Wall et al. where a significant difference between patient-group and healthy-group was only observed three hours after exercise [15]. In the current study, a significant difference between the groups was only observed at day 180. Thus, pimobendan might support the oxygenation of the tissue and lower anerobic metabolism by inotropic effects. This possible positive influence of administering pimobendan was only observed at day 180, however. Ferasin et al. observed a coincided decrease in plasma lactate concentration with better exercise tolerance of dogs with acquired heart failure (New York Heart Association (NYHA) classes 2 and 3) [26]. Thus, a lower increase during exercise and lower level of lactate after exercise might indicate a better exercise tolerance and thus a better life-quality of the dogs treated with pimobendan. Research shows that people with chronic obstructive lung disease as well as healthy people have lower lactate values for several weeks after exercise or respiratory endurance training [27, 28]. Thus, a decrease in lactate values after exercise, observed in the current study, might be a sign of more exercise tolerance because of more exercise over several weeks at home in the form of exercise training.

### Questionnaire

Another important item rarely examined in prospective studies is the influence of pimobendan on dogs’ quality of life, an important factor for dog owners in deciding on a possible therapy for their dogs [8]. Boswood et al. detected no differences in quality of life between dogs treated with pimobendan or a placebo at day 35 [29]. However, in the current study, dogs treated with pimobendan were significantly more active than dogs treated with a placebo at day 180 of administering pimobendan. Nevertheless, two patient owners from the placebo-group indicated that their dogs were more active as well. This result shows that a double-blinded study design is important to objectify the patient owners’ limited ability to judge as well as the results of a questionnaire. The difference in the findings between the current study and the study by Boswood et al. could be due to the different point of time of the evaluation or due to differences in the owner questionnaires used. However, more activity does not mean a prolonged survival time or stability of the disease. Nonetheless, it can be an indicator of better quality of life. Thus, the current study suggests that some dogs with mitral valve disease stage ACVIM B1 treated with pimobendan show more activity than before initiating the treatment. In the future, this finding can be helpful for patient owners to decide whether they would like their dogs to be treated with pimobendan in early stages of mitral valve disease [8].

### Echocardiographic parameters

In the current study, no reduction in heart size or rise in fractional shortening was observed due to treatment with pimobendan in dogs with mitral valve disease without cardiomegaly. There were no negative effects detected in echocardiography in dogs treated with pimobendan. Chetboul et al. observed that dogs treated with pimobendan had more severe mitral valve lesions than those dogs treated with benazepril after 512 days of treatment [6]. Size and velocity of mitral valve regurgitation increased in the pimobendan-group but not in the benazepril-group. In the current study, no changes in mitral valve morphology were seen in the control examinations. Since no quantification of size and velocity of mitral regurgitation were performed in the follow-up-examinations in the current study, no statement can be made regarding possible changes in mitral regurgitation in the Doppler examination. Boswood et al. observed that dogs with asymptomatic mitral valve disease with cardiomegaly (i. e., in the more advanced stage ACVIM B2) have a reduced heart size after treatment with pimobendan [29]. It is also possible that in the current study, no differences were observed because a lower number of dogs, in comparison to the study of Boswood et.al, were examined. The current study was a pilot study and further studies with a higher number of patients are needed. Thus, there is no consensus regarding effects of pimobendan on cardiac morphological changes.

### Limitations

An important limitation of the study was the inclusion of dogs of different breeds and sizes. As reported by Swimmer et al., physical characteristics like leg length or dog weight have only low to moderate correlation with results in exercise testing [30]. Since there was no significant difference between dogs in both groups regarding weight and age, we expect the influence of physical characteristics of the dogs on results of exercise testing to be minimal.

In addition to this, the observation time in the study was short so that no data regarding time before onset of clinical symptoms or survival time were collected. Both parameters are important factors in clinical decision-making in patients with preclinical mitral valve disease. To examine a possible positive effect of administering pimobendan on disease progression and survival time in dogs with asymptomatic mitral valve disease without cardiomegaly, larger studies are needed.

## Conclusion

Standardized submaximal exercise testing in conjunction with measurement of lactate was evaluated as a possible tool for monitoring the fitness-level and the oxygenation of the peripheral tissue in dogs with preclinical mitral valve disease and as a method for monitoring cardiac therapy in dogs with early stage mitral valve disease. Echocardiographic parameters were not influenced by pimobendan in this study. Some dogs with early stage mitral valve disease might benefit from a therapy with pimobendan, as shown by higher levels of activity reported by the owners compared to those animals without treatment. Research with a larger number of patients and inclusion of time to onset of symptoms and survival time are needed to further assess the effect of pimobendan in patients with preclinical mitral valve disease without cardiomegaly.

## Supporting information

S1 Questionnaire (DOCX)

S1 Fig (TIFF)

S2 Fig (PNG)

S1 File (DOCX)
