# Supplementary figures and images for: Effect of pimobendan on physical fitness, lactate and echocardiographic parameters in dogs with preclinical mitral valve disease without cardiomegaly
Source: PLoS One. 2019 Oct 3;14(10):e0223164. doi: 10.1371/journal.pone.0223164 (PMC6776412; doi:10.1371/journal.pone.0223164)

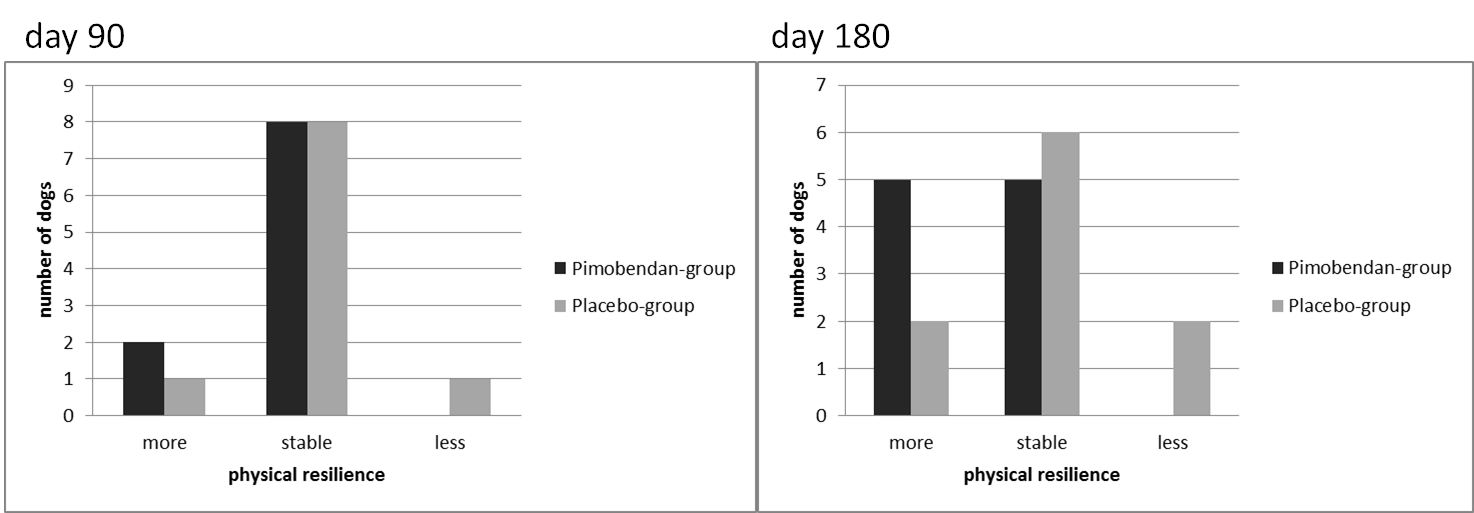

Supplement: S1 Fig — (TIFF) [file pone.0223164.s002.tiff]

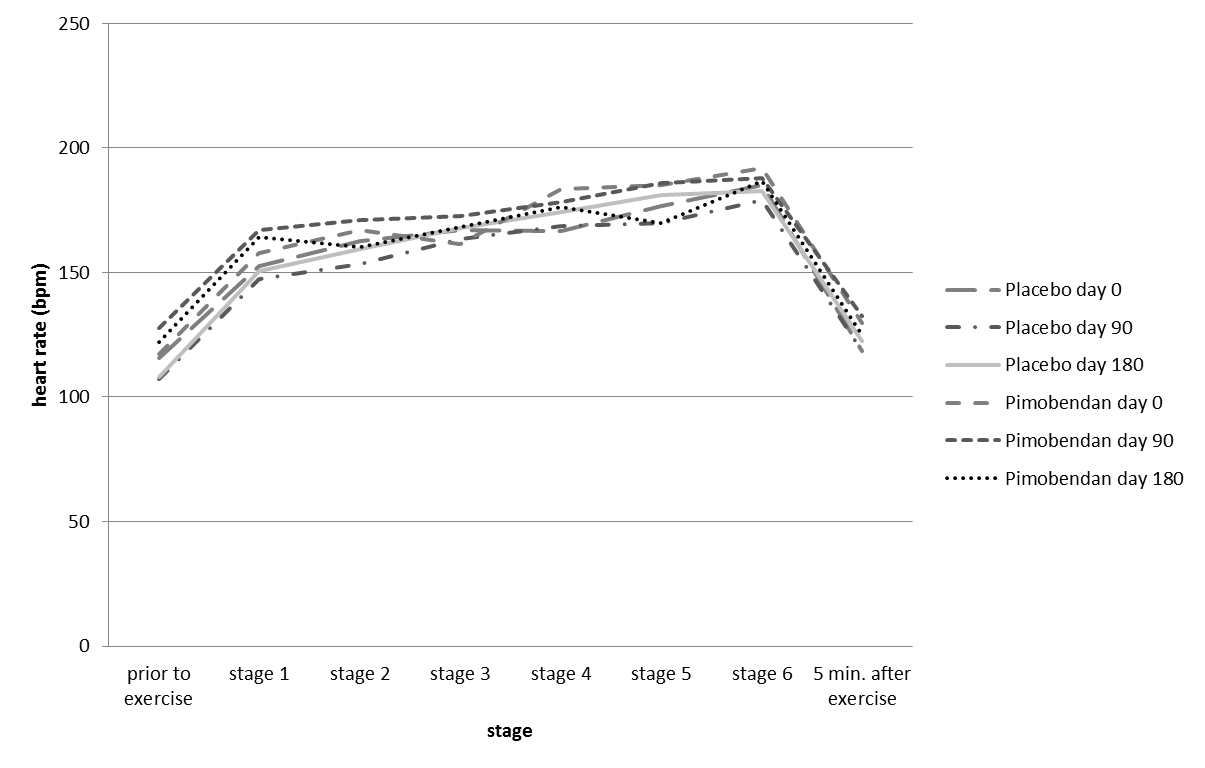

Supplement: S2 Fig — (PNG) [file pone.0223164.s003.png]
