# Supplementary material for: Effect of pimobendan on physical fitness, lactate and echocardiographic parameters in dogs with preclinical mitral valve disease without cardiomegaly
Source: PLoS One. 2019 Oct 3;14(10):e0223164. doi: 10.1371/journal.pone.0223164 (PMC6776412; doi:10.1371/journal.pone.0223164)
Supplement: S1 File — (DOCX) [file pone.0223164.s004.docx]

**S1 Fig: Changes in physical resilience (more, stable, less) evaluated by patient owners.**

The number of dogs is labeled on the y-axis and the changes during 90 or 180 days of administering medication (more, stable, less) are mentioned on the x-axis. The pimobendan-group is marked in black and the placebo-group in gray.

**S2 Fig: Mean heart rate (bpm) during the standardized submaximal exercise test of the pimobendan- and placebo-group**.

The heart rate (bpm) is indicated on the y-axis and the point of measuring during the standardized submaximal exercise test is given on the x-axis. The pimobendan- and placebo-group at days 0, 90 and 180 have different colors and lines, which is seen on the right side of the figure.
